# Automated three-dimensional radiomic body composition analysis enhances survival prediction in resectable non‑small cell lung cancer

**DOI:** 10.1186/s41747-026-00802-2

**Published:** 2026-09-18

**Authors:** Yilong Huang, Chuanpu Li, Fan Yang, Xin Chen, Xiaobo Chen, Yanqi Huang, Zhenguang Zhang, Lei Yang, Yuanming Jiang, Hanxue Cun, Zhanglin Mou, Wei Yang, Zaiyi Liu, Bo He

**Affiliations:** 1 Department of Medical Imaging, The First Affiliated Hospital of Kunming Medical University, Kunming, China; 2 Department of Radiology, Guangdong Provincial People’s Hospital (Guangdong Academy of Medical Sciences), Southern Medical University, Guangzhou, China; 3 Guangdong Provincial Key Laboratory of Artificial Intelligence in Medical Image Analysis and Application, Guangzhou, China; 4 School of Biomedical Engineering, Southern Medical University, Guangzhou, China; 5 Guangdong Provincial Key Laboratory of Medical Image Processing, Guangzhou, China; 6 The First School of Clinical Medicine, Zhejiang Chinese Medical University, Hangzhou, China; 7 Department of Radiology, Guangzhou First People’s Hospital, School of Medicine, South China University of Technology, Guangzhou, China; 8 Department of Radiation Oncology (Maastro), GROW Research Institute for Oncology and Reproduction, Maastricht University Medical Centre+, Maastricht, The Netherlands

**Keywords:** Artificial intelligence, Body composition, Non-small cell lung cancer, Radiomics, Survival prediction

## Abstract

**Objective:**

To develop and validate a machine learning radiomics model integrating automated three-dimensional body composition and tumor imaging features for predicting overall survival in resectable non–small cell lung cancer (NSCLC).

**Materials and methods:**

This multicenter retrospective study included patients with resectable NSCLC treated between January 2013 and December 2017, who were assigned to training, internal, and external validation cohorts. A fully automated deep learning algorithm was developed for body composition segmentation. Radiomic features from tumor and body composition were extracted and integrated using extreme gradient boosting. Model performance was assessed using the concordance index (C-index) and time-dependent area under the curve (AUC), with interpretability evaluated by SHapley Additive exPlanations (SHAP). Kaplan–Meier analysis was performed for survival stratification.

**Results:**

Among 1,038 patients (mean age, 61.8 ± 10.7 years; 58.66% male), 293 (28.2%) died over a median follow-up of 3.31 years. In the training cohort, both tumor score and body composition score were independently associated with overall survival (hazard ratio 2.72 and 2.03, respectively; all *p* < 0.001). Incorporating body composition radiomics significantly improved discrimination compared with tumor-only models across cohorts (all *p* < 0.05). The comprehensive model, integrating clinicopathological factors, tumor score, and body composition score, demonstrated strong predictive capability for 1, 2, 3, and 5-year survival (AUCs > 0.80). SHAP analysis identified tumor score and body composition score as dominant predictors, stratifying patients into four phenotypes with distinct prognoses (all log-rank *p* < 0.05).

**Conclusion:**

Integrating automated three-dimensional body composition with tumor radiomics enhances survival prediction and provides incremental value for postoperative risk stratification in resectable NSCLC.

**Key Points:**

***Question*** Standard staging for resectable non-small cell lung cancer lacks objective three-dimensional quantification of host body composition, thereby limiting individualized prognostic assessment.

***Findings*** Machine learning models integrating automated three-dimensional body composition and tumor radiomics significantly outperform conventional tumor imaging in overall survival prediction.

***Relevance statement*** Automated three-dimensional body composition radiomics integrated with tumor imaging improves survival prediction in resectable non-small cell lung cancer, enabling more precise postoperative risk stratification and supporting individualized follow-up and supportive care strategies.

**Graphical Abstract:**

**Figure:**
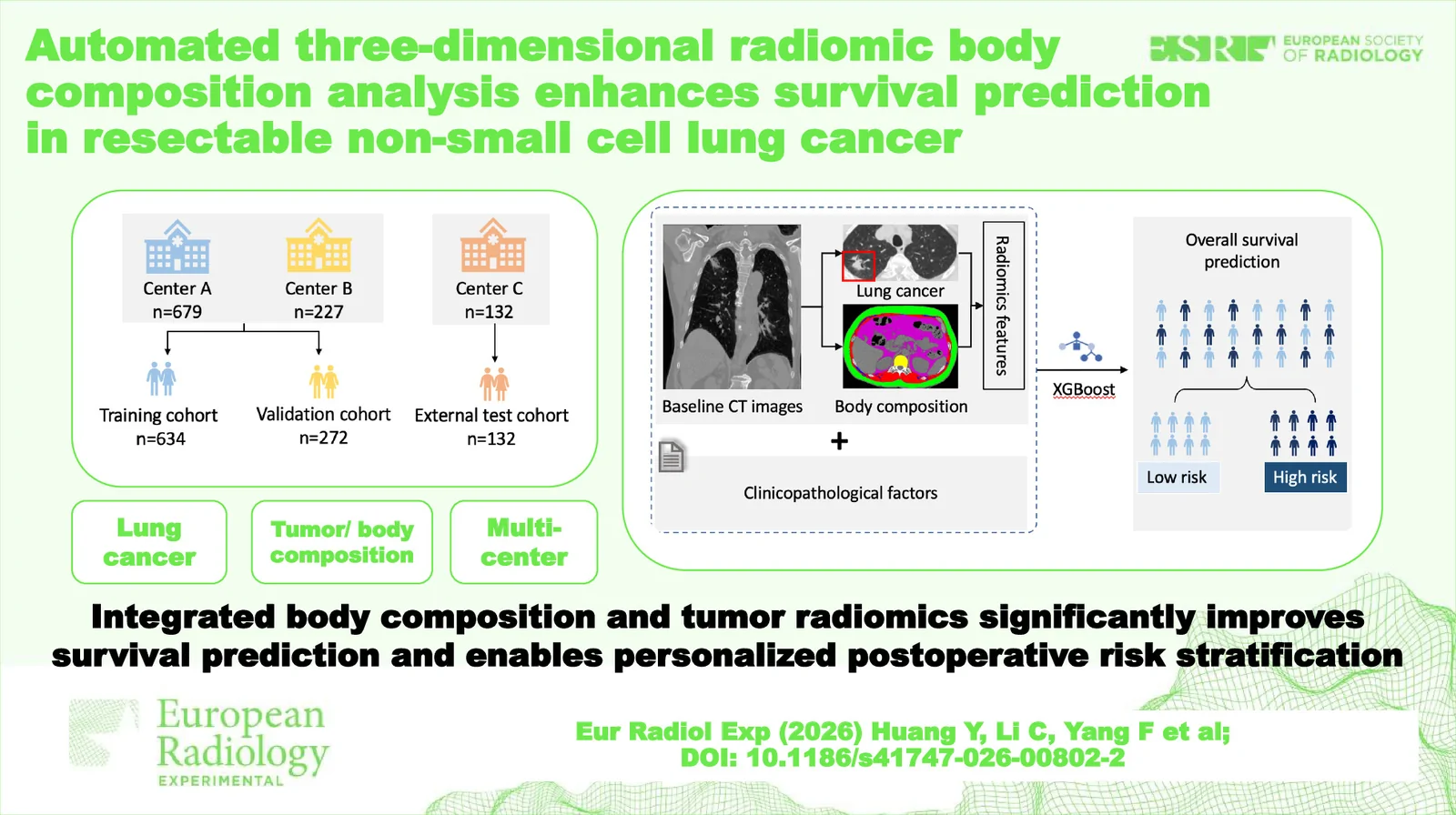

## Introduction

Non–small-cell lung cancer (NSCLC) exhibits substantial survival heterogeneity even after curative-intent resection, underscoring the limitations of conventional tumor node metastasis staging for individualized risk stratification [1, 2]. Although radiomics has enabled high-throughput quantification of tumor phenotypes from CT images [3, 4], survival prediction models derived solely from intratumoral features have demonstrated only modest discriminatory performance, suggesting that tumor-centric imaging biomarkers incompletely capture the biological determinants of outcome [5, 6].

Beyond lung cancer lesions, body composition emerges as a critical, modifiable, yet underexplored prognostic factor in cancer outcomes [7–9]. Traditional body composition metrics, such as skeletal muscle (SM) and adipose tissue distributions, have been increasingly recognized as potential predictors of clinical outcomes, including chemotherapy toxicity, surgical complications, and reduced OS [10–12]. However, current CT-based body composition assessment remains methodologically constrained: most studies rely on manual or semi-automated segmentation, two-dimensional single-slice analysis, and limited quantitative descriptors [12, 13], thereby failing to comprehensively characterize the three-dimensional, heterogeneous, and spatially complex nature of body composition.

Although some studies have made initial attempts to explore the clinical value of radiomics-based body composition analysis, several limitations persist [14–17]. These studies often restrict analysis to two-dimensional images [14, 16], rely on manual vertebrae localization and body composition segmentation [14, 16, 17], and fail to comprehensively assess body composition components [14–17]. Furthermore, these models based on body composition often omit crucial tumor-specific features and lack interpretability [14–17].

We hypothesized that a fully automated, three-dimensional deep learning framework integrating spine-guided body composition segmentation with tumor radiomics could provide a more comprehensive imaging phenotype for survival prediction in resectable NSCLC. To test this hypothesis, we developed and multicentrically validated an end-to-end deep learning model enabling automated lumbar spine localization and three-dimensional segmentation of major body composition components on baseline chest CT. Using radiomics features derived from both tumor and segmented body composition, we constructed an interpretable machine learning model to predict overall survival and evaluated its incremental prognostic value beyond clinicopathological factors and tumor-only radiomics signatures. This integrative, automated approach aims to bridge systemic host phenotypes and tumor characteristics, advancing imaging-based precision prognostication in surgically treated NSCLC.

## Materials and methods

### Patients

**Fig. 1:**
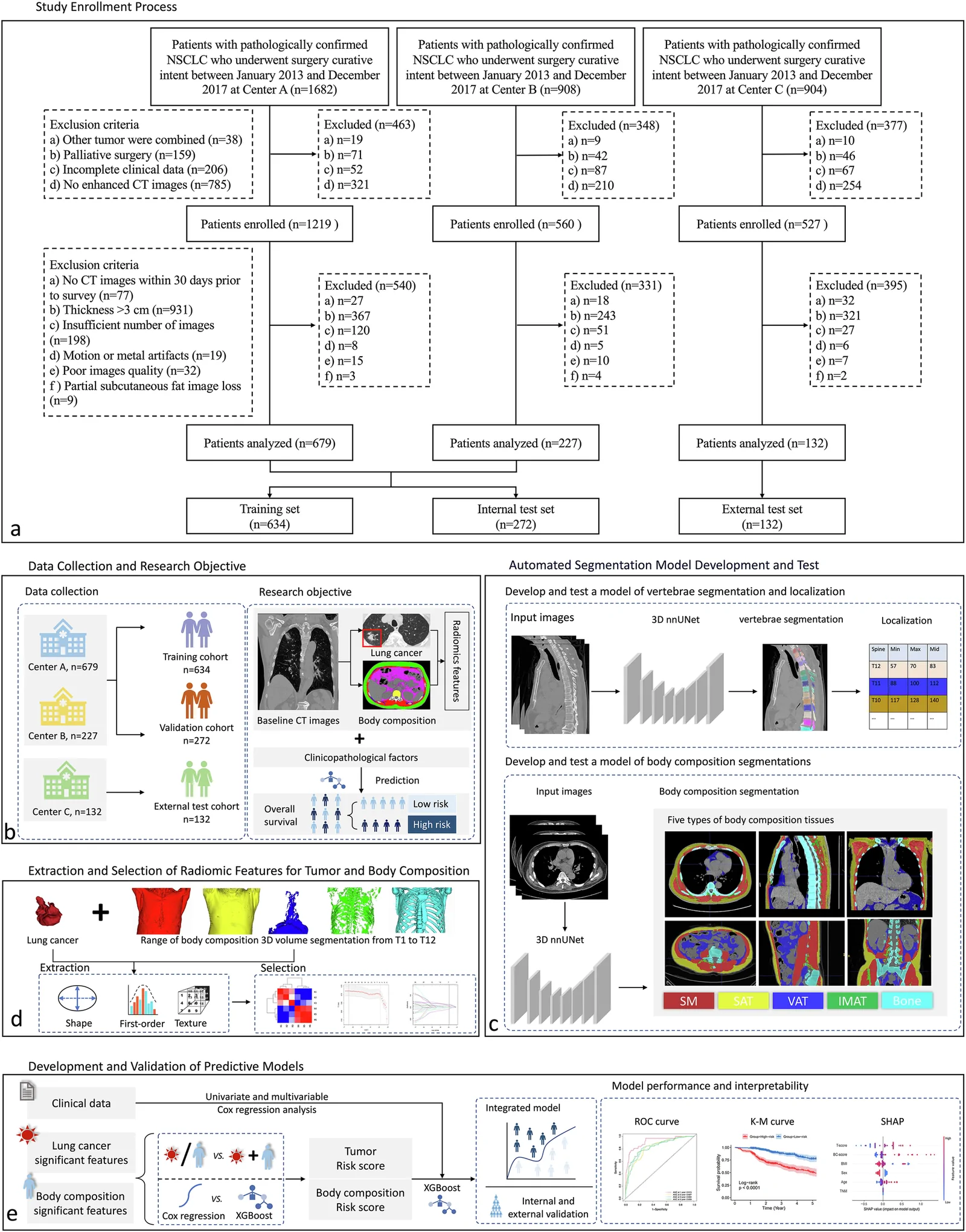
Study workflow overview. **a** Flowchart of the study enrollment process. **b** Data collection and research objective. **c** Automated segmentation model development and test. **d** Extraction and selection of radiomic features for tumor and body composition. **e** Development and validation of predictive models. NSCLC, Non-small-cell lung cancer; CT, Computed tomography; SM, Skeletal muscle; SAT, Subcutaneous adipose tissue; VAT, Visceral adipose tissue; IMAT, Intermuscular adipose tissue; ROC, Receiver operating characteristic; K-M, Kaplan–Meier

This retrospective multicenter study obtained ethical approval from the Ethics Committee and Institutional Review Boards of Guangdong Provincial People’s Hospital (No. KY-Z-2021-030-02; June 8, 2021), Guangzhou First People’s Hospital (No. S-2023-052-01; November 7, 2023), and First Affiliated Hospital of Kunming Medical University (No. 2024-L-283; November 21, 2024). The study adhered to the Declaration of Helsinki. Between January 2013 and December 2017, consecutive patients who underwent curative-intent surgical resection for pathologically confirmed NSCLC at three tertiary medical centers were screened. The inclusion criteria were: (1) histologically confirmed diagnosis of NSCLC, (2) contrast-enhanced chest CT scans performed within 30 days prior to surgery, and (3) complete clinicopathological and follow-up data available. The exclusion criteria were as follows: (1) had a history of other malignancies, and (2) the quality of CT images or the slice thickness did not meet the requirements. Detailed recruitment procedures are provided in Fig. 1a. A total of 2,880 patients were screened, of whom 1,038 met the inclusion criteria and were included in the final analysis. Patients from Centers A and B were randomly allocated in a 7:3 ratio into training and internal validation cohorts. The independent cohort from Center C was reserved for external validation of the survival prediction models. The sample size estimation was calculated based on the reported hazard ratio [5, 6] as described in Supplementary Method 1. The overall study design is illustrated in Fig. 1b–e.

### Clinicopathological data and patients’ outcome

We retrospectively collected clinicopathological data for all eligible patients, including age, sex, height, weight, body mass index (BMI), smoking status, surgery date, histological subtype, TNM staging (8th edition, 2017), history of malignancy, family history, and chemotherapy. OS was defined as the time from the initial surgery to the date of death from any cause or the last follow-up in October 2023, whichever occurred first. Survival data were obtained through telephone follow-ups and electronic medical records.

### Image acquisition and tumor volume segmentation

All patients underwent contrast-enhanced chest CT scans within 1 month prior to surgery. Details of the CT scanning protocols and image preprocessing are provided in Supplementary Method 2 and Table S1. To mitigate center effects from CT images acquired across different scanners and hospitals, all original CT images underwent preprocessing. Initially, the images were resampled to 1 × 1 × 1 mm³ using a linear interpolation algorithm to standardize voxel spacing. Subsequently, a 25 HU bin width was applied to discretize voxel intensity, thereby reducing noise. Primary tumor segmentations were manually annotated on venous-phase enhanced CT images by two radiologists (Y.L.H. and X.C.) with 6 and 10 years of CT imaging experience, respectively. The radiologists were blinded to all clinicopathological data and used ITK-SNAP software (version 3.8.0, http://www.itksnap.org/) for annotation. A senior radiologist (B.H.) subsequently reviewed all volume of interest and made adjustments as needed to resolve discrepancies.

### Automated vertebral localization and body composition segmentation

Automated vertebral localization and body composition segmentation were performed using the AutoPanoM framework (v1.0.0.0, MediaLab, Guangzhou, China) [18]. The system localized the T1–T12 vertebrae to guide the volumetric segmentation of skeletal muscle, subcutaneous adipose tissue, visceral adipose tissue, intermuscular adipose tissue, and bone. The comprehensive analysis workflow and model architecture are depicted in Figs. 1c, S1, and S2. Detailed protocols for model training and evaluations of segmentation performance across cohorts are provided in Supplementary Methods 3–5.

### Radiomic feature extraction and selection

Radiomics features were extracted from tumor and segmented body composition volumes using PyRadiomics (version 3.1.0) [19]. These features encompassed shape descriptors, first-order statistics, and texture metrics. Comprehensive details regarding the feature extraction parameters are provided in Supplementary Methods 6. Non-biological batch effects were detected between these three datasets using principal component analysis and corrected using ComBat [20]. For feature selection, all radiomics features were standardized using z-scores. Furthermore, we performed univariate Cox regression analysis on all features, applying a significance threshold of *p* < 0.05. Next, Spearman correlation analysis was conducted on the remaining features. Features with an absolute correlation coefficient exceeding 0.8 were considered redundant and excluded from further analysis. Finally, the remaining features underwent Least Absolute Shrinkage and Selection Operator (LASSO) Cox regression to identify the most predictive features for survival outcomes.

### Machine learning model development and validation for survival prediction

Two radiomic risk scores, the tumor score (T-score) and the body composition score (BC-score), were derived from the features selected by LASSO-Cox regression. We then pursued a stratified modeling approach to predict poor overall survival. Initially, a set of models (Clinic, T-Rad, BC-Rad, TB-Rad) was developed using Cox regression to establish baseline performance. To enhance prognostic accuracy by accounting for potential non-linear relationships and complex interactions, we further developed two integrated models using the extreme gradient boosting (XGBoost) algorithm: a clinical-tumor (CT) model and a comprehensive clinical-tumor-body composition (CTB) model.

Subsequently, candidate clinical factors underwent univariate Cox analysis, and features with *p* < 0.05 were included in a multivariate Cox analysis to construct a clinical prognostic model. The identified clinical factors, along with T-score and BC-score, were then integrated using the XGBoost algorithm. The SHAP was applied to the constructed XGBoost model to quantify the contribution of individual features to each patient’s prognostic assessment. Based on the absolute SHAP values of the T-score and BC-score, if the value of |T-score| is higher, it is classified as the Tumor type; if the value of |BC-score| is higher, it is classified as the BC type. Subsequently, depending on the sign of the higher SHAP value, a positive value indicates the harmful type, while a negative SHAP value indicates the conservative type. Eventually, four categories are obtained, namely Tumor-harmful, Tumor-conservative, BC-harmful, and BC-conservative.

### Statistical analysis

Statistical analyses were performed with R software (version 4.2.2). Continuous variables were compared using the Student’s *t*-test or Mann–Whitney *U*-test, while categorical variables were analyzed using the chi-square test or Fisher’s exact test, as appropriate. The concordance index (C-index) was used to evaluate model performance, with comparisons between different models conducted using bootstrap resampling. For survival analysis, univariate and multivariate Cox proportional hazards regression analyses with bidirectional stepwise selection were performed. To control for multiple testing in secondary analyses, the Bonferroni correction method was applied. Cutoff values were determined in the training cohort and applied unchanged to validation cohorts. Kaplan–Meier (K-M) curves were plotted and compared using log-rank test. Time-dependent receiver operating characteristic (ROC) analysis was performed to estimate 1-, 2-, 3-, and 5-year AUC values. A *p*-value < 0.05 was considered indicative of statistical significance.

## Results

### Clinicopathological characteristics

Baseline clinicopathological characteristics of the 1,038 patients with NSCLC are summarized in Table 1. The entire cohort consisted of patients with a mean age of 61.81 ± 10.69 years, including 608 males (58.7%). By the end of the follow-up period, 172 patients (25.3%) from Center A, 83 (36.6%) from Center B, and 38 patients (28.8%) from Center C had died. The median follow-up times were 2.86 years for Center A, 2.43 years for Center B, and 7.16 years for Center C. Significant differences were observed among the three centers in variables such as age, sex, BMI, history of malignancy, family history, histological type, TNM staging, and chemotherapy (*p* < 0.05). Figure S3 shows the results after batch effect detection and correction. In the training cohort, age, male, low BMI, and higher TNM stage were associated with shorter OS (Table 2). Following univariate and multivariate Cox regression analyses, age, sex, BMI, and TNM stage were retained as significant predictors.

**Table 1:** Characteristics of patients in three centers

Characteristics	Center A (*N* = 679)	Center B (*N* = 227)	Center C (*N* = 132)	*p*-value
Age (years), Median (IQR)	61 (54, 68)	65 (58, 70)	64 (57, 71)	< 0.001*
Sex				0.022*
Male	391 (57.58%)	149 (65.64%)	68 (51.52%)	
Female	288 (42.42%)	78 (34.36%)	64 (48.48%)	
BMI (kg/m^2^), Median (IQR)	22.49 (20.26, 24.45)	21.04 (20.00, 23.48)	21.94 (20.30, 24.63)	< 0.001*
Smoking status				0.325
Yes	459 (67.60%)	146 (64.32%)	95 (71.97%)	
No	220 (32.40%)	81 (35.68%)	37 (28.03%)	
Histological type				< 0.001*
Adenocarcinoma	516 (76.00%)	183 (80.62%)	132 (100%)	
Non-adenocarcinoma	163 (24.00%)	44 (19.38%)	0 (0%)	
TNM				< 0.001*
I	437 (64.36%)	134 (59.03%)	101 (76.52%)	
II	93 (13.70%)	27 (11.89%)	13 (9.85%)	
III	149 (21.94%)	66 (29.07%)	18 (13.64%)	

*p*-values were calculated using the Kruskal–Wallis test for continuous variables and the chi-square test or Fisher’s exact test for categorical variables, as appropriate

*BMI* Body mass index, *TNM* Tumor node metastasis, *IQR* Interquartile range

* *p*-value < 0.05

**Table 2:** Univariate and multivariable Cox regression analysis for selecting clinical features in the training cohort

Characteristics	Univariate analysis		Multivariate analysis	
	HR (95% CI)	*p*-value	HR (95% CI)	*p*-value
Age	1.03 (1.02–1.04)	< 0.001*	1.04 (1.02–1.05)	< 0.001*
Sex (female)	0.45 (0.31–0.63)	< 0.001*	0.53 (0.37–0.75)	< 0.001*
BMI (kg/m^2^)	0.96 (0.94–0.98)	< 0.001*	0.96 (0.95–0.98)	< 0.001*
Smoking status (yes)	1.75 (1.30–2.34)	< 0.001*		
Histological type (non-adenocarcinoma)	0.68 (0.44–1.04)	0.076		
TNM				
II	2.91 (1.90–4.46)	< 0.001*	3.01 (1.96–4.62)	< 0.001*
III	4.65 (3.31–6.54)	< 0.001*	4.51 (3.19–6.37)	< 0.001*

Only variables identified as significant (* represents *p* < 0.05) in the univariable analyses were entered into the multivariable analysis. *p*-values in multivariable Cox regression were adjusted using the Bonferroni correction for multiple comparisons. All reported associations remained statistically significant after correction

*HR* Hazard ratio, *CI* Confidence interval, *BMI* Body mass index, *TNM* Tumor node metastasis

### Automated vertebral localization and body composition segmentation performance

The automated framework achieved robust performance for vertebral localization and body composition segmentation across multiple centers. Vertebral localization accuracy reached 97.5% (194/200) in the internal validation cohort. For body composition segmentation, Dice coefficients exceeded 0.98 for skeletal muscle, subcutaneous adipose tissue, visceral adipose tissue, and bone, while the most challenging compartment, intermuscular adipose tissue, achieved a Dice coefficient of 0.895 (Table S2). Similar performance was observed in the external validation cohort, demonstrating good generalizability (Table S3). Furthermore, automated measurements showed excellent agreement with manual reference standards, with both correlation coefficients and intraclass correlation coefficients exceeding 0.99 across all body composition compartments (Table S4). Representative segmentation results are shown in Figs. S4 and S5. Additional analyses of segmentation performance and failure cases are provided in Supplementary Results 1.

### The performance and interpretability of the survival prediction model

**Fig. 2:**
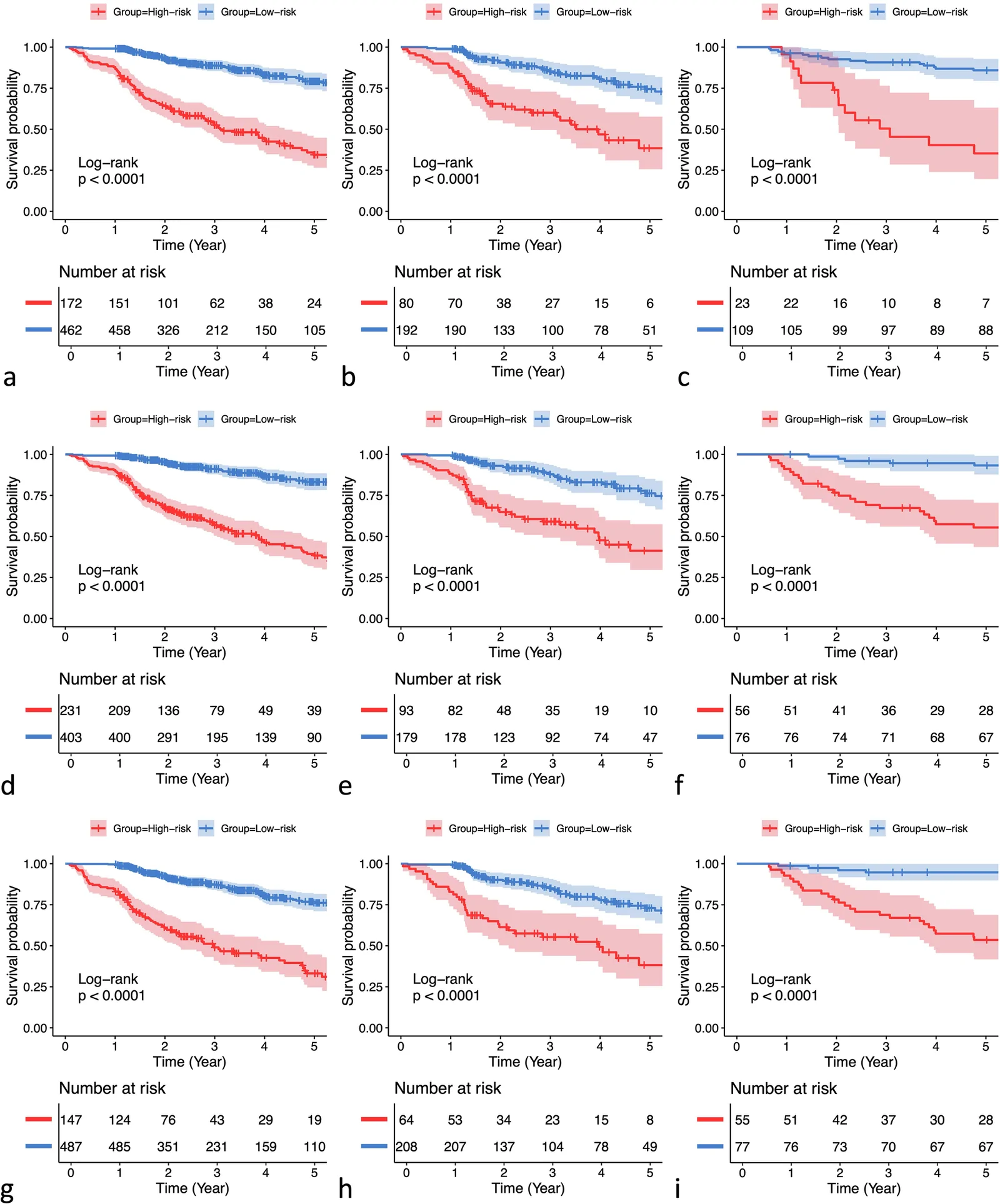
Kaplan–Meier survival curves for overall survival stratified by risk groups from XGBoost models incorporating clinical, tumor, and body composition features. **a**–**c** the Clinical model; **d**–**f** the CT model (clinical + tumor radiomics); and **g**–**i** the CTB model (clinical + tumor + body composition radiomics). **a**, **d**, **g** Training cohort. **b**, **e**, **h** Internal validation cohort. **c**, **f**, **i** External validation cohort

Both T-score and BC-score demonstrated significant associations with OS in the training cohort based on multivariable Cox regression analysis, yielding hazard ratios of 2.72 (95% CI: 2.17–3.41) and 2.03 (95% CI: 1.84–2.24), respectively. K-M curves analysis indicated that higher BC-score and T-score were significantly associated with worse OS in the training, internal validation, and external validation cohorts (Figs. 2 and S6). The log-rank tests confirmed statistically significant differences between prognostic groups (all log-rank test *p* < 0.001). The TB-Rad model outperformed the T-Rad model, with significantly higher C-index values in both internal and external validation cohorts (0.749 and 0.742 *versus* 0.695 and 0.692, respectively; all *p* < 0.05; Table S5).

**Fig. 3:**
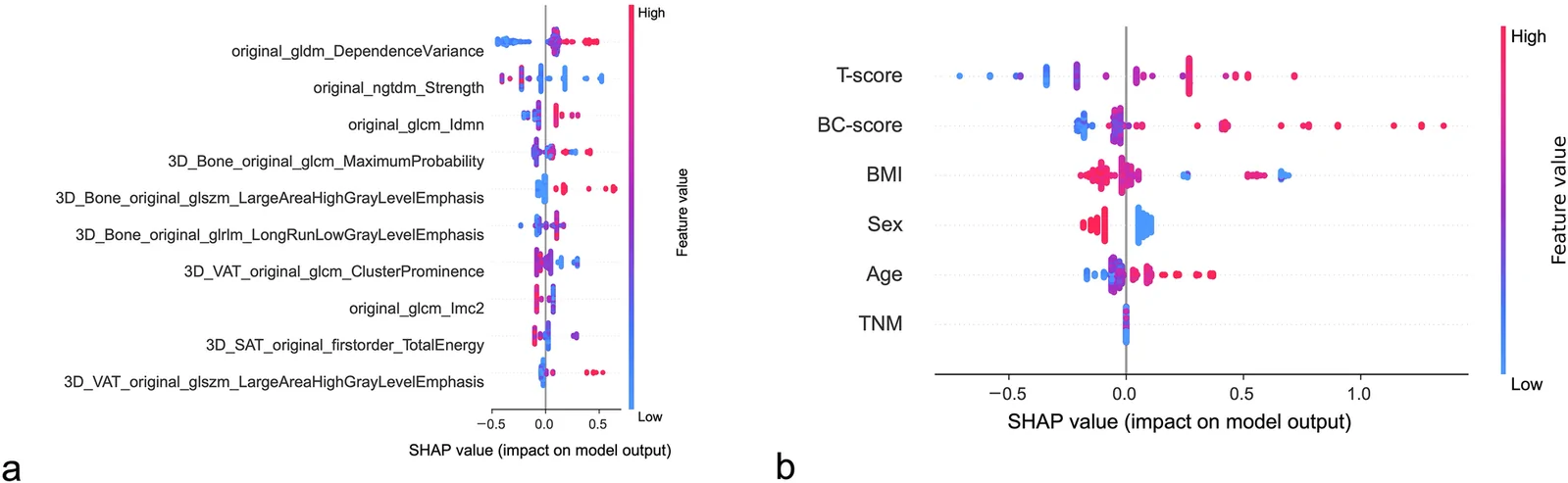
XGBoost model constructed based on clinical, tumor, and body composition features. **a** SHAP beeswarm plot for the model integrating tumor and body composition radiomics (TB-Rad model). **b** SHAP beeswarm plot for the comprehensive model integrating clinical, tumor, and body composition features (CTB model). T-score, Tumor score; BC-score, Body composition score; BMI, Body mass index; TNM, Tumor node metastasis

We used SHapley Additive exPlanations (SHAP) values to interpret XGBoost models and find key predictive features. SHAP Beeswarm plots in Figs. 3a and S7 visualize the relative importance of tumor and body composition radiomics features, providing insights into the key predictive mechanisms driving survival predictions.

### Clinical comprehensive model performance

**Fig. 4:**
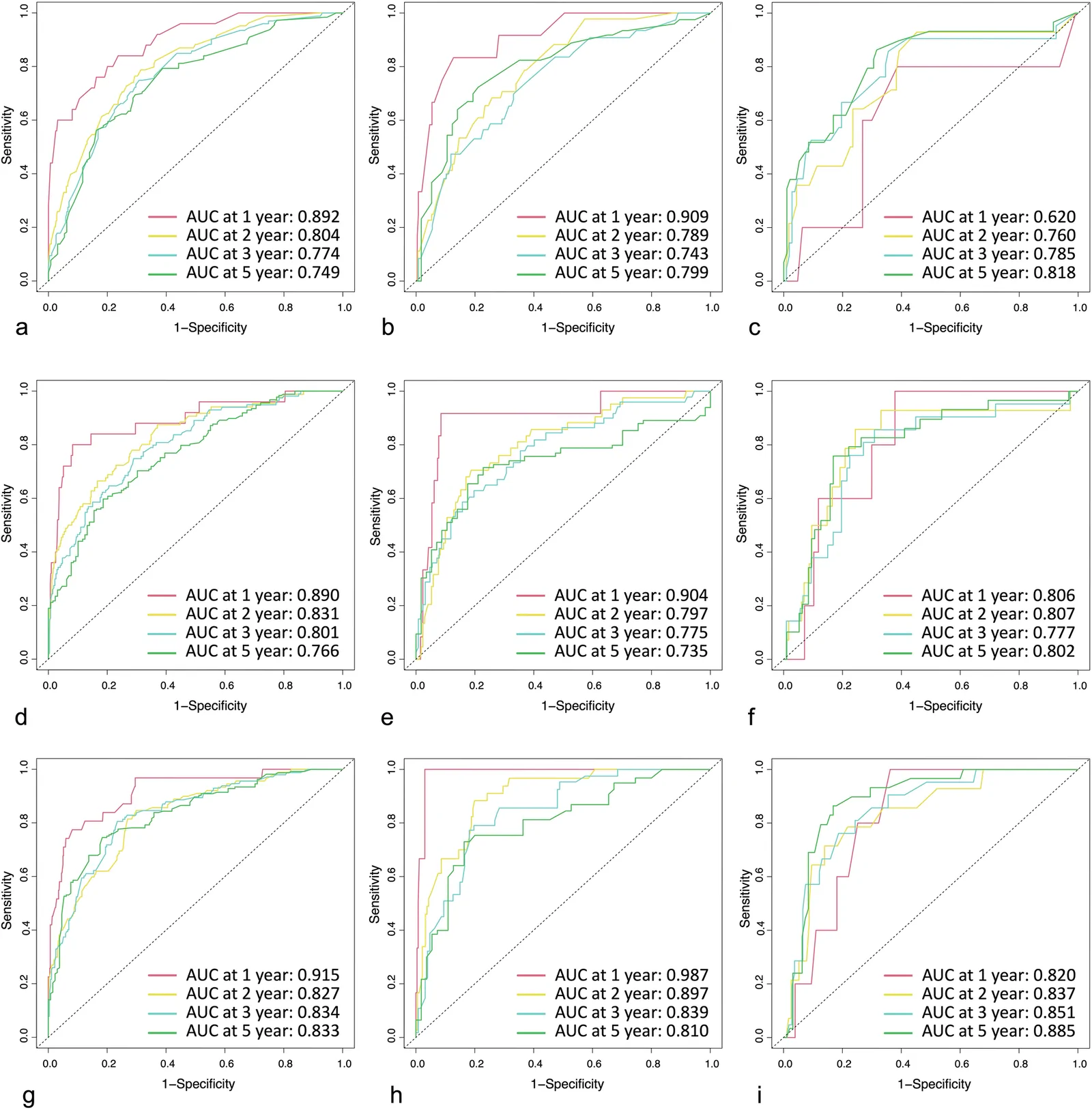
Receiver operating characteristic curves of the XGBoost models for predicting 1-, 2-, 3-, and 5-year survival. The AUCs were calculated for each cohort and time point. **a**–**c** Clinic model. **d**–**f** Combined model integrating clinical and tumor features (CT model). **g**–**i** Combined model integrating clinical, tumor lesion, and body composition features (CTB). **a**, **d**, **g** Training cohort. **b**, **e**, **h** Internal validation cohort. **c**, **f**, **i** External validation cohort. AUC, Area under the curve

The CTB model successfully stratified patients into distinct risk groups, as visualized in K-M curves in Fig. 2. The XGBoost-derived CTB model exhibited superior prediction performance compared to the CT model in both the internal (C-index: 0.780 *versus* 0.725) and external (0.779 *versus* 0.726) validation cohorts (Table 3). As shown in Fig. 4, the CTB model exhibited strong capability in predicting 1-, 2-, 3-, and 5-year survival, with time-dependent AUC values all above 0.80. Moreover, the CTB model achieved higher AUC values than the CT model for predicting survival at all these time points in both validation cohorts, and these improvements were statistically significant at every time point in the internal validation cohort (all *p* < 0.05). Furthermore, subgroup analyses demonstrated that the CTB model generally maintained its robust prognostic stratification capability across most TNM stages (I–II and III) and N-stage subgroups. Although the log-rank test did not reach statistical significance in the N stage 0–1 internal validation cohort (*p* = 0.25), likely due to the limited sample size and event rate within this specific subgroup, the model successfully distinguished between high- and low-risk patients in all other clinical contexts evaluated (Fig. S8). Decision curve analysis demonstrated that the CTB model yielded superior net clinical benefit compared to the clinical and CT models across a wide range of threshold probabilities in the training, internal validation, and external validation cohorts (Fig. S9).

**Table 3:** Survival prediction performance of different models

Models	Training cohort		Internal validation cohort		External validation cohort	
	C-index (95% CI)	*p*-value	C-index (95% CI)	*p*-value	C-index (95% CI)	*p*-value
Cox regression						
Clinic model	0.645 (0.554, 0.723)	-	0.632 (0.487, 0.724)	-	0.636 (0.452, 0.814)	-
CT model	0.723 (0.645, 0.782)	< 0.001*	0.687 (0.585, 0.792)	0.005*	0.713 (0.558, 0.847)	< 0.001*
CTB model	0.764 (0.721, 0.793)	0.041^#^	0.726 (0.673, 0.771)	0.042^#^	0.745 (0.661, 0.827)	0.039^#^
XGBoost						
Clinic model	0.673 (0.616, 0.767)	-	0.643 (0.518, 0.767)	-	0.671 (0.520, 0.823)	-
CT model	0.754 (0.738, 0.810)	< 0.001*	0.725 (0.664, 0.790)	< 0.001*	0.726 (0.654, 0.807)	< 0.001*
CTB model	0.795 (0.759, 0.831)	0.035^#^	0.780 (0.720, 0.820)	0.022^#^	0.779 (0.710, 0.848)	0.036^#^

*CI* Confidence interval, *CT model* Model integrating clinical variables and tumor radiomics score, *CTB model* Model integrating clinical variables, tumor radiomics score, and body composition radiomics score

* Compared with the clinic model, *p* < 0.05

^#^ Compared with the CT model, *p* < 0.05

Further SHAP analysis of the CTB model revealed that the T-score and BC-score were the most influential features driving the model’s predictions (Fig. 3b). Moreover, an analysis of the differences in T-score and BC-score across different TNM stages is provided in Fig. S10.

### Phenotypic characterization

**Fig. 5:**
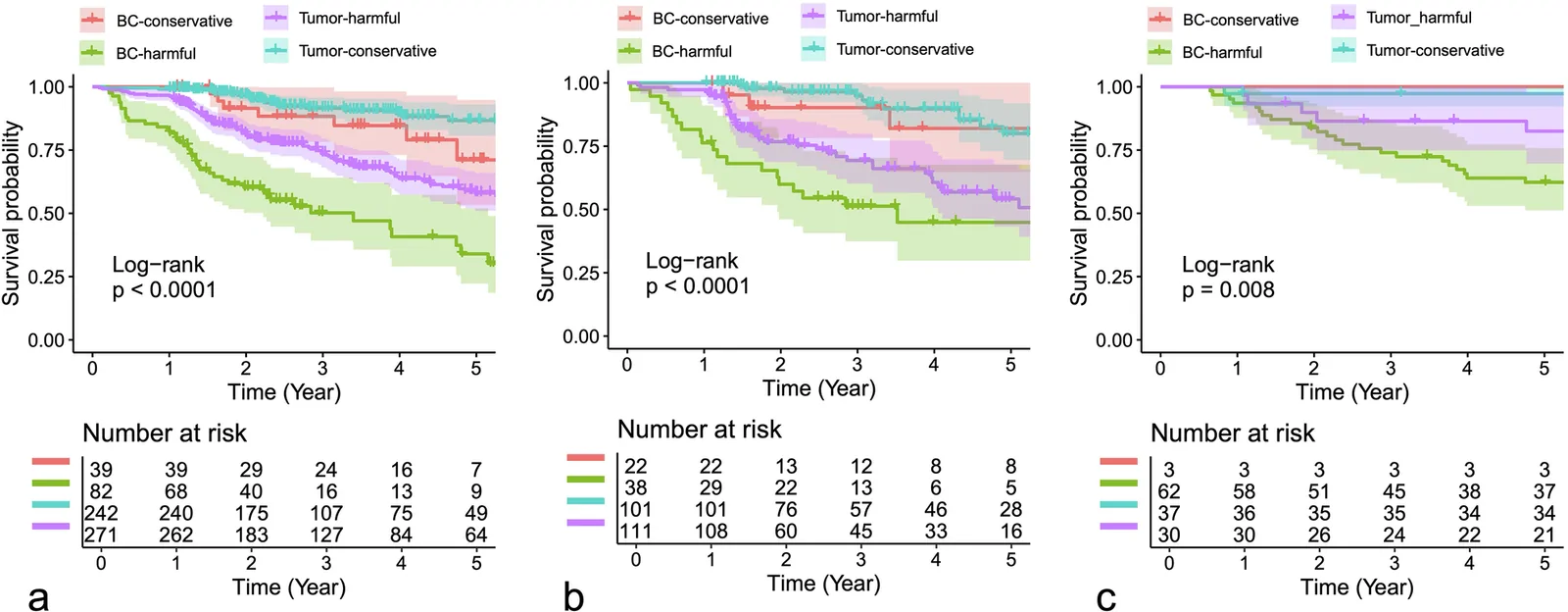
The Kaplan–Meier curves of the composite phenotype by tumor and body composition scores. **a** Training cohort. **b** Internal validation cohort. **c** External validation cohort. BC, Body composition

By combining the T-score and BC-score, we identified four distinct phenotypes with significant differences in prognostic outcomes (Fig. 5). The tumor-conservative type exhibited the best prognosis. In contrast, the BC-harmful type consistently demonstrated the poorest prognosis across all three cohorts, even surpassing the tumor-harmful type in terms of adverse survival outcomes. The statistical significance of these prognostic differences was confirmed by the log-rank test, which yielded *p*-values < 0.001 for all cohorts.

## Discussion

In this multicenter study, we developed and externally validated an interpretable survival prediction framework integrating tumor radiomics, automated three-dimensional body composition radiomics, and clinicopathological factors for patients with resectable NSCLC. The resulting model demonstrated robust prognostic performance across independent validation cohorts and consistently outperformed tumor-only approaches, highlighting the incremental value of host body composition information for individualized risk stratification. To enable large-scale and reproducible extraction of body composition phenotypes, we incorporated a fully automated vertebral localization and segmentation framework, which achieved high accuracy and excellent agreement with expert annotations across multiple centers.

Body composition is increasingly recognized as a determinant of clinical outcomes; however, its implementation in routine CT analysis has been limited by the lack of accurate and fully automated segmentation tools. Recent advances in deep learning have improved automation [21], yet most prior approaches relied on two-dimensional imaging or semi-automated workflows. Our model leverages fully automated three-dimensional CT analysis with integrated vertebral localization to enable standardized volumetric quantification across tissue compartments [22]. In external validation, segmentation performance for skeletal muscle, subcutaneous adipose tissue, and visceral adipose tissue was robust and reproducible. Notably, intermuscular adipose tissue, historically difficult to delineate, was segmented with high accuracy, representing improved segmentation consistency compared with prior two-dimensional approaches [23–25]. Across all components, automated measurements demonstrated strong agreement with manual reference standards, supporting the reliability and scalability of the proposed framework.

This multicenter study integrates three-dimensional radiomics analysis of both tumor and body composition for survival prediction in resectable NSCLC. Prior investigations largely relied on two-dimensional single-slice measurements at the L3 level and evaluated limited body composition metrics [14, 15, 26]. By extracting multidimensional radiomics features from volumetric tumor and systemic compartments, our approach provides a more comprehensive characterization of disease biology and host status. Ten radiomics features, each for tumors and body composition, were selected and used to develop predictive models, employing both traditional statistical methods (Cox regression) and machine learning (XGBoost). The results indicated that independent tumor or body composition models could assess survival risk in patients with NSCLC across different cohorts. In the performance analysis, the C-index of the tumor Cox regression model improved after incorporating body composition radiomics features. While an earlier small-scale study suggested the potential complementary value of body composition radiomics, their generalizability was limited [15]. Furthermore, our approach of combining the T-score and BC-score into four phenotypes effectively stratified survival risk. Across all three cohorts, the BC-harmful type consistently demonstrated the shortest survival time, demonstrating comparably poor or even worse survival. After integrating clinical data, tumor, and body composition risk scores, the model achieved the highest C-index in the internal and external validation cohorts, and successfully predicted 1-, 2-, 3-, and 5-year survival, with all AUC values exceeding 0.80. Collectively, these findings support the integration of body composition radiomics with tumor characteristics to enhance individualized prognostic assessment.

Tumor radiomics signatures have demonstrated prognostic value in NSCLC [6, 27]; however, heterogeneity in study design has resulted in variable feature composition and limited biological integration. In contrast, our study incorporated a comprehensive three-dimensional radiomics signature of body composition, extending prognostic assessment beyond tumor-centric phenotyping. Feature attribution analysis indicated that radiomics metrics derived from SAT, VAT, IMAT, and bone contributed meaningfully to survival prediction. Nevertheless, elucidating the intricate relationships between biological processes and radiomics features of body composition remains a major challenge, with limited targeted research available. Referring to previous studies on traditional body composition analysis and tumor prognosis, cancer patients with higher SAT content have a longer survival period [28], and SAT has also been incorporated as an important potential factor into the prediction model [17, 29]. In this study, the “Total_Energy” of SAT was related to volume, providing energy storage and anti-tumor effect. The “Kurtosis” may reflect extreme metabolic states such as obesity or emaciation, both of which are associated with poor prognosis [30]. Conversely, excessive VAT may contribute to chronic inflammation, thereby accelerating tumor progression [31]. IMAT was often not routinely evaluated due to the difficulty of segmentation. Interestingly, this study found that the “Cluster_Prominence” of IMAT is associated with survival outcomes. “Cluster_Prominence” quantifies the prominence of localized clustering patterns within grayscale distributions, providing insights into structural heterogeneity. A high “Cluster_Prominence” of IMAT may indicate excessive intermuscular fat infiltration, reduced muscle quality, and conditions such as muscle atrophy, cachexia, and metabolic dysfunction—factors known to negatively impact cancer survival. This is supported by a prior systematic review linking myosteatosis to poor overall survival in colorectal cancer patients [32]. Additionally, we conducted a novel analysis of bone radiomics features, revealing their unexpected but biologically plausible association with prognosis. Four features indicative of bone texture uniformity and continuity were incorporated into our model. Existing research has revealed systemic crosstalk between lung cancer and bone [33, 34]. Lung cancer can remotely stimulate osteoblast activity even in the absence of metastasis. In turn, these osteoblasts supply neutrophil recruitment, which promotes cancer progression. The tumor radiomics score directly reflects tumor heterogeneity and biological aggressiveness, making it a primary determinant of prognosis. These mechanistic interpretations remain hypothesis-generating and warrant further biological validation. Meanwhile, the body composition radiomics score may impact survival through mechanisms involving metabolism, inflammation, immune status, and physiological reserves. Our results highlight that integrating tumor and body composition radiomics provides a multidimensional biological basis for prognostic assessment, enhancing predictive accuracy for NSCLC survival outcomes. Furthermore, although the workflow is fully automated, its real-world implementation and clinical utility have not yet been prospectively evaluated. Future studies should assess workflow integration, computational efficiency, user acceptance, and clinical impact in routine practice settings.

This study still has several limitations. Firstly, as a retrospective multicenter study, it is susceptible to potential selection and inherent biases. Although prospective longitudinal studies offer a more ideal design, the long wait time required to assess survival outcomes for resectable lung cancer makes such studies challenging. Additional external validation cohorts would further strengthen the generalizability of the model. Second, while OS is the most definitive and clinically relevant endpoint in oncologic studies, it is influenced by multiple non-imaging factors, including adjuvant treatment strategies, comorbidities, and underlying molecular tumor heterogeneity. These factors may introduce residual confounding that cannot be fully accounted for in imaging-based models. Accordingly, the proposed radiomics signatures should be interpreted as complementary prognostic biomarkers reflecting imaging-derived tumor and host phenotypes, rather than as standalone determinants of survival outcomes. Finally, the predictive performance of the survival model could be further refined by integrating additional established prognostic factors, including, but not limited to, epidermal growth factor receptor mutation status and programmed death-ligand 1 expression levels.

In conclusion, this study introduces and validates a comprehensive three-dimensional body composition segmentation model based on automated vertebral localization. Additionally, the integration of tumor and body composition image data into a machine learning radiomics model shows significant potential for survival prediction in patients with resectable NSCLC. Radiomics-based body composition analysis provides important incremental value for postoperative survival risk assessment in patients.

## Supplementary information

**Additional file1: Fig. S1** Architecture of the automated model structure for vertebral localization. **Fig. S2** Architecture of the 3D automated model structure for comprehensive body composition segmentation. **Fig. S3** Principal component analysis scatter plots of patients from three centers. (a) Before batch correction. (b) After batch correction. **Fig. S4** Representative visualizations of automated spine localization and body composition segmentation. (a) Automated localization of vertebral bodies. (b-d) Exemplary results of automated segmentation for the five body composition components. **Fig. S5** Representative cases of automatic body composition segmentation. The original images (scans) are followed by images of SAT, muscle, IMAT, VAT, and bone. For each scan, the first row shows the segmentation using the manual method (Reference); the second row shows the prediction by 3D nnU-Net. Arrows indicate areas of annotation differences. **Fig. S6** Kaplan–Meier curves of overall survival based on body composition and tumor lesion Cox regression models. (a-c) Tumor radiomics (T-Rad) model. (d-f) Body composition radiomics (BC-Rad) model. (g-i) Integrated tumor and body composition (TB-Rad) model. (a, d, g) Training cohort. (b, e, h) Internal validation cohort. (c, f, i) External validation cohort. **Fig. S7** SHAP beeswarm plots illustrating feature importance in XGBoost models. (a) Model based on tumor radiomics features. (b) Model based on body composition radiomics features. **Fig. S8** Kaplan–Meier overall survival curves stratified by the CTB model in TNM subgroups and N-stage subgroups of TNM stage III. (a-c) TNM I-II stage. (d-f) TNM III stage. (g-h) N stage 0-1. (i-j) N stage 2-3. (a, d, g, i) Training cohort. (b, e, h, j) Internal validation cohort. (c, f) External validation cohort. CTB model, model integrating clinical variables, tumor radiomics score, and body composition radiomics score. **Fig. S9** Decision curve analysis evaluating the clinical utility of the predictive models. (a) Training cohort. (b) Internal validation cohort, and (c) External validation cohort. **Fig. S10** Box plots of Tumor-score and Body composition-score in different TNM stages. (a) Tumor-score (T-score). (b) Body composition-score (BC-score). (c) Combined T-score and BC-score. **Table S1** CT scanning parameters. **Table S2** Performance of the body composition automatic segmentation model in the internal validation cohort. **Table S3** Performance of the body composition segmentation model in the external validation cohort. **Table S4** Correlation and consistency analysis of the segmentation of body composition by manual and automated model. **Table S5** Survival prediction performance of radiomics-based models.

## Data Availability

The data that support the findings of this study are available from the corresponding author, Bo He (email: kmmu_hb@163.com), upon reasonable request. The data are not publicly available since this could compromise the privacy of research participants.

## Abbreviations

AUC: Area under the curve
BC: Body composition
BMI: Body mass index
C-index: Concordance index
CT: Computed tomography
CTB: Clinical-tumor-body composition model
IMAT: Intermuscular adipose tissue
K-M: Kaplan–Meier
NSCLC: Non–small cell lung cancer
OS: Overall survival
SAT: Subcutaneous adipose tissue
SHAP: SHapley Additive exPlanations
TNM: Tumor-node metastasis
VAT: Visceral adipose tissue
XGBoost: Extreme gradient boosting
