# Supplementary material for: Automated three-dimensional radiomic body composition analysis enhances survival prediction in resectable non‑small cell lung cancer
Source: Eur Radiol Exp. 2026 Sep 18;10:135. doi: 10.1186/s41747-026-00802-2 (PMC13588999; doi:10.1186/s41747-026-00802-2)
Supplement: Supplementary file 1 [file 41747_2026_802_MOESM1_ESM.pdf]

Automated Three-dimensional Radiomic Body Composition Analysis Enhances Survival Prediction in Resectable Non-Small Cell Lung Cancer

ELECTRONIC SUPPLEMENTARY MATERIAL

Supplementary Methods

Supplementary Results

Supplementary Figures

Supplementary Tables

Eur Radiol Exp (2026) Huang Y, Li C, Yang F et al.

Supplementary Methods

1. Sample size estimation

The sample size for this retrospective study was calculated using Power Analysis and Sample Size (PASS) software (version 21.0.3). A two-sided log-rank test was employed for survival sample size estimation, with a significance level (α) of 0.05, a statistical power (1-β) of 0.90, and inclusion of patients with complete follow-up. Based on a hazard ratio (HR) of 1.77 for the radiomics signature, the minimum required sample size was determined to be 129 patients, including 64 in the control group (low-risk group) and 65 in the treatment group (high-risk group). In this study, a total of 634 patients were enrolled in the training set, 272 in the internal test set, and 132 in the external test set, which exceeded the calculated sample size requirement and ensured sufficient statistical power.

2. CT image acquisition and Image preprocessing

All patients underwent routine contrast-enhanced chest CT. Although the CT scan protocols across the three centers were similar, slight variations in scanning systems and parameters existed. Detailed acquisition parameters are provided in Supplementary Table 1. Axial CT images were retrieved from the Picture Archiving and Communication System (PACS). To address data variability caused by differences in instruments and parameters across the multicenter cohort, all CT images were standardized.

During body composition segmentation, the images were initially resampled to a voxel resolution of 0.8625 × 0.8625 × 2 mm³ to optimize memory usage. The resampled scans were then cropped into image blocks of 256 × 256 × 96 voxels. For the body composition prediction phase, a two-stage normalization process was applied to prepare the data for analysis and reduce inter-cohort variability. First, the CT images were rescaled to a uniform voxel size of 1 × 1 × 1 mm³ using cubic spline interpolation. Second, pixel intensity values were standardized to a consistent range of -1024 to 1024 Hounsfield Units (HU) and adjusted to a standard mediastinal imaging window with a window level of 50 and a window width of 350.

3. Structure of automated model for vertebral localization and comprehensive body composition segmentation

The U-Net encoder consists of five layers, each containing two convolutional blocks. Each block includes a 3D convolution, instance normalization, and the Leaky ReLU activation function. The number of channels in the encoder layers is 32, 64, 128, 256, and 320, respectively. All 3D convolutions use a kernel size of 3×3×3. The strides for the first convolutional block in the five layers are 1×1×1, 2×2×2, 2×2×2, 2×2×2, and 1×2×2, respectively, while the strides for the second convolutional block in each layer are 1×1×1.

The decoder mirrors the encoder with five layers. The bottom-most layer uses a single 3D transposed convolution with 320 channels, a kernel size of 1×2×2, and a stride of 1×2×2. The other four layers each contain two convolutional blocks followed by a 3D transposed convolution. These layers have 256, 128, 64, and 32 channels, respectively. The convolutional blocks in these layers use a kernel size of 3×3×3 and a stride of 1×1×1, while the 3D transposed convolutions use a kernel size of 2×2×2 with a stride of 2×2×2 for up-sampling. At the end of the network, the Sigmoid activation function is applied to output class scores for each pixel. The final segmentation is performed by assigning each pixel to the tissue class with the highest score. Prior to training, image preprocessing included windowing adjustments. The model was trained using a combination of Dice loss and cross-entropy loss functions. Optimal hyperparameters were determined experimentally, with a batch size of 2 and an initial learning rate of 0.01, which was reduced using a polynomial decay function. The Adam optimizer was employed. The code was written in Python 3.9.0, using the PyTorch 2.0.0 backend. Model training was conducted on a system with 128 GB of RAM, utilizing an RTX 4090 GPU with 24 GB of VRAM.

4. Development and validation of automated vertebral localization and comprehensive body composition segmentation model

To address the challenges of manual segmentation and enhance accuracy, we developed an artificial intelligence model to automate vertebral segmentation and

positioning, followed by full-body composition segmentation. This included vertebrae, SM, SAT, VAT, and IMAT. The model was built using the nnUNet framework (https://github.com/MIC-DKFZ/nnUNet), employing its 3D full-resolution adaptive medical image segmentation method. The segmentation model was trained on 1000 cases from Centers A and C, and validated on independent internal (n=200, Centers A and C) and external (n=200, Center B) datasets. A musculoskeletal radiologist (ZGZ), with 15 years of experience, reviewed all vertebral localization results to assess accuracy and analyze causes of misalignment. Importantly, the segmentation model and the prognostic prediction models were developed as independent analytical tasks. Segmentation model training was performed solely using anatomical image data and pixel-level segmentation annotations, without access to survival outcomes, radiomic features, or prognostic labels. Consequently, segmentation model development was entirely separated from radiomic feature selection, prognostic model training, and survival prediction validation.

5. Evaluation of segmentation performance and generalizability

The vertebral and body composition segmentation results generated by the model were compared with those of an internal validation set (n = 200) manually corrected by a musculoskeletal radiologist with 15 years of experience (ZGZ). The performance was analyzed using the Dice score, Jaccard score, true positive rate, false positive rate, and the difference in the total partition area calculated from the segmentation. For regions A and B, the Dice score is defined as $2*|A\cap B|/|A + B|$, and the Jaccard score is defined as $|A\cap B|/|A\cup B|$. To evaluate the ability of our automated method to generalize to other datasets, the segmentation performance of the model on 200 patients from the Center B dataset was analyzed. The prediction results were compared with the manually corrected segmentations. The comparisons were carried out over the entire volume of the images.

6. Radiomic feature

Radiomics features were extracted using PyRadiomics version 3.1.0, an open-source Python package. For each 3D-segmented tissue type, including tumors, SM, IMAT,

SAT, VAT and bone, all features available in PyRadiomics were automatically extracted. These features encompassed shape descriptors, first-order statistics, and texture metrics such as gray-level co-occurrence matrix (GLCM), gray-level size zone matrix (GLSZM), gray-level run length matrix (GLRLM), neighborhood gray-tone difference matrix (NGTDM), and gray-level dependence matrix (GLDM).

Supplementary Results

1. Detailed evaluation of segmentation performance

Representative examples of vertebral localization and body composition segmentation are shown in Figures S4 and S5. The automated framework successfully identified vertebral landmarks and segmented five body composition compartments, including skeletal muscle, subcutaneous adipose tissue, visceral adipose tissue, intermuscular adipose tissue, and bone.

Among the 200 cases in the internal validation cohort, vertebral mislocalization occurred in four patients, including two cases with sacralized lumbar vertebrae, one case with severe metal artifacts, and one case with marked scoliosis. Despite these challenging anatomical variations, overall localization accuracy remained high. Among all segmented compartments, IMAT represented the most challenging tissue class and showed lower Dice performance and greater variability than skeletal muscle and adipose tissue compartments, likely reflecting its relatively small volume and indistinct tissue boundaries. Nevertheless, segmentation accuracy remained acceptable and reproducible across cohorts. Detailed quantitative performance metrics for internal validation, external validation, and agreement analyses are provided in Tables S2–S4.

Supplementary Figures

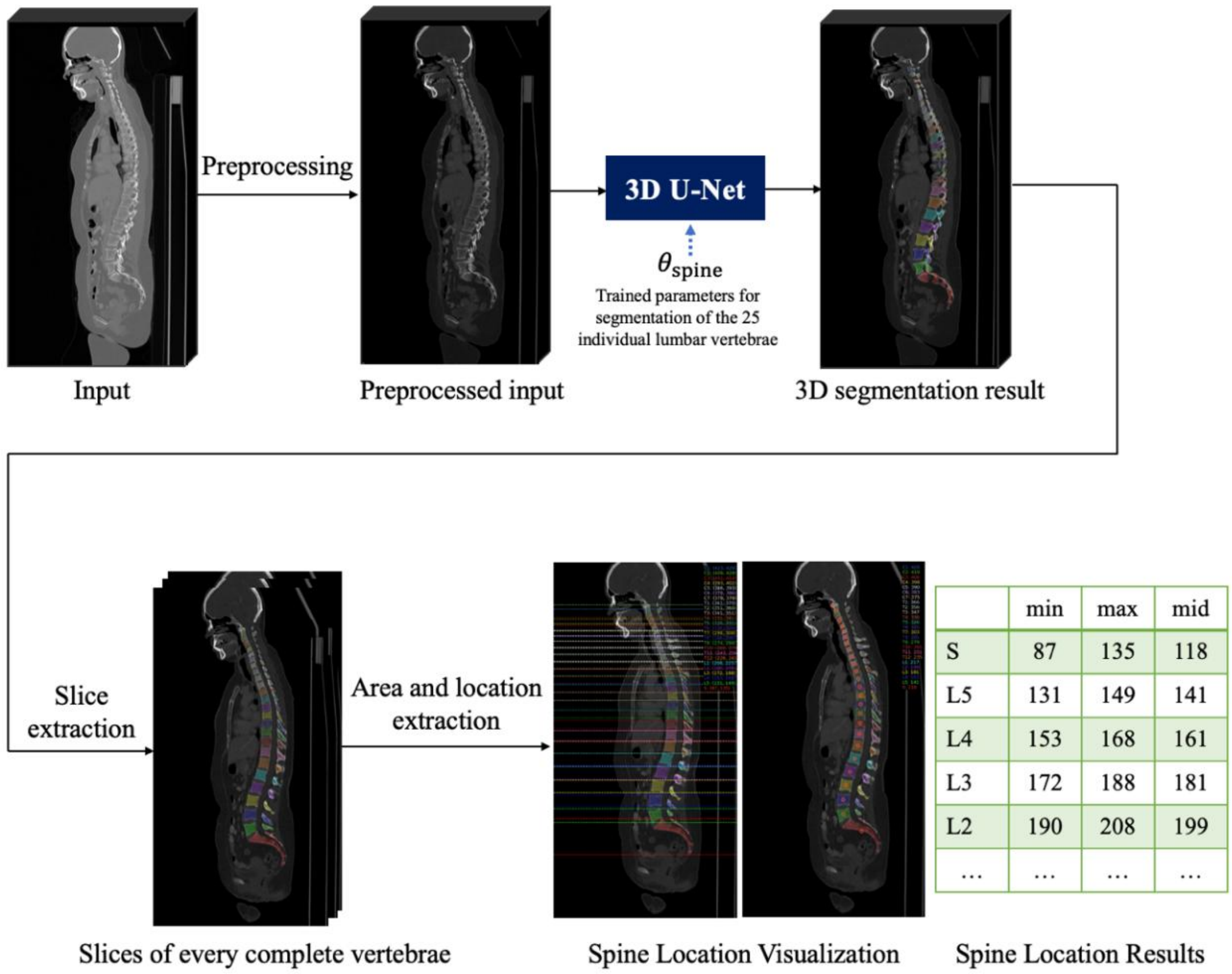

	min	max	mid
S	87	135	118
L5	131	149	141
L4	153	168	161
L3	172	188	181
L2	190	208	199
…	…	…	…

Fig. S1 Architecture of the automated model structure for vertebral localization.

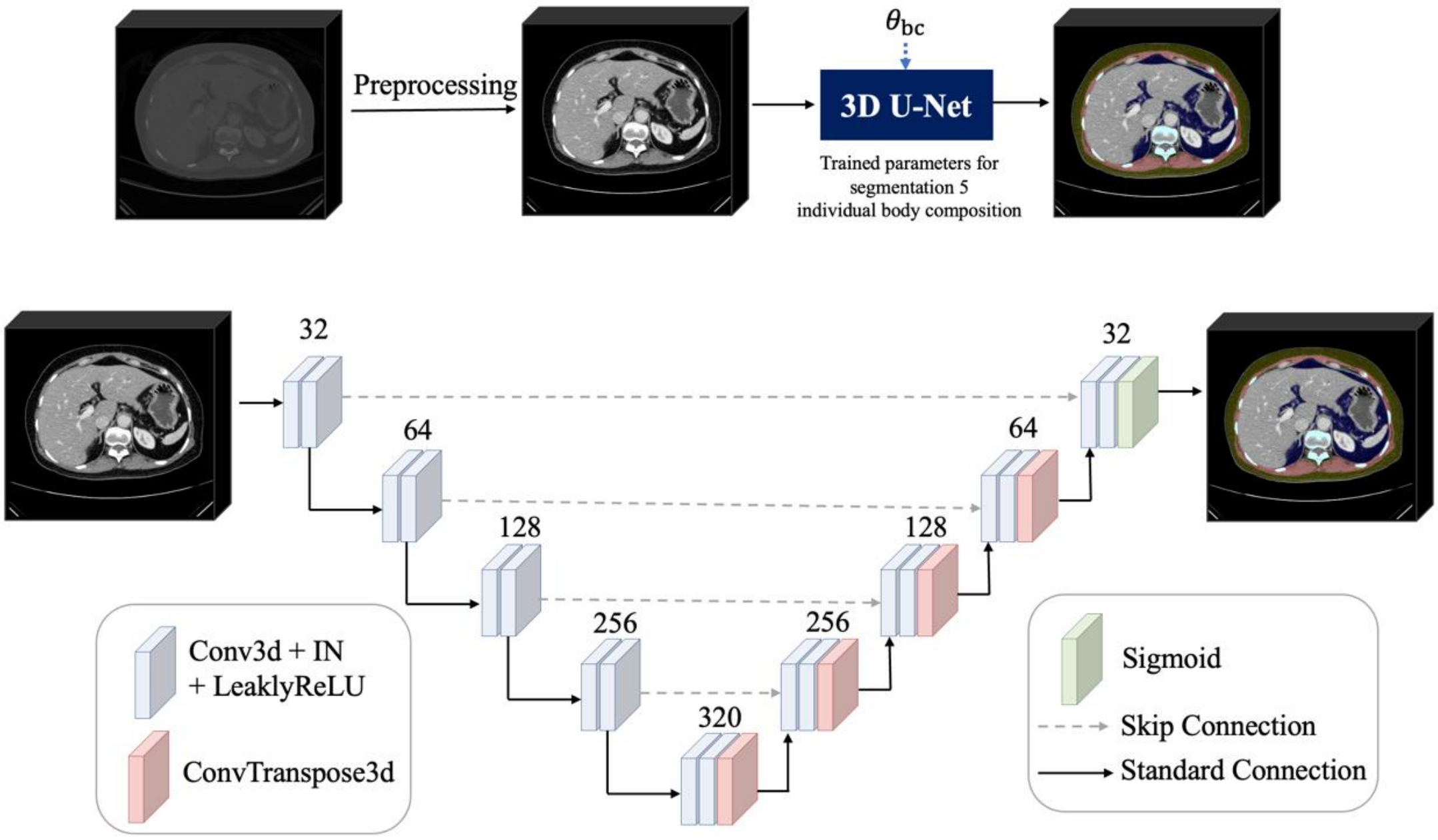

Fig. S2 Architecture of the 3D automated model structure for comprehensive body composition segmentation.

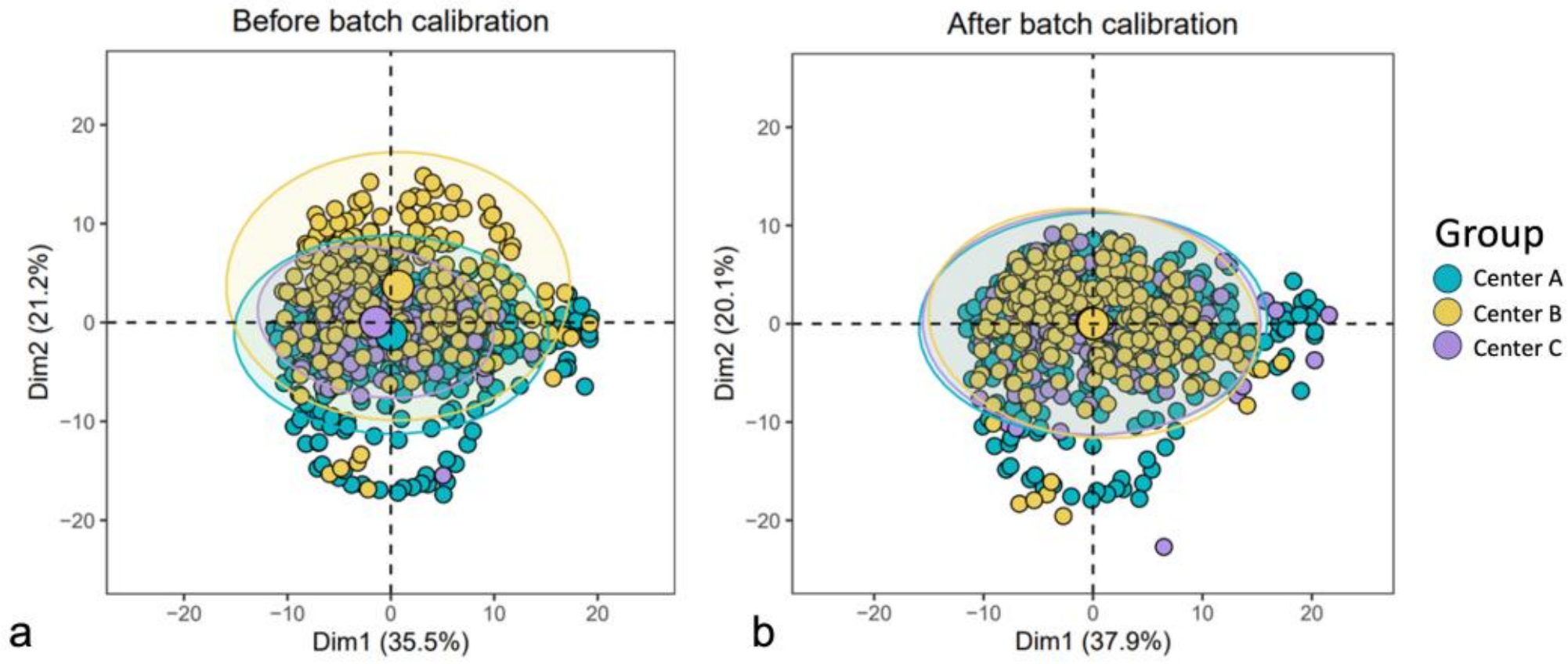

Fig. S3 Principal component analysis scatter plots of patients from three centers. (a) Before batch correction. (b) After batch correction.

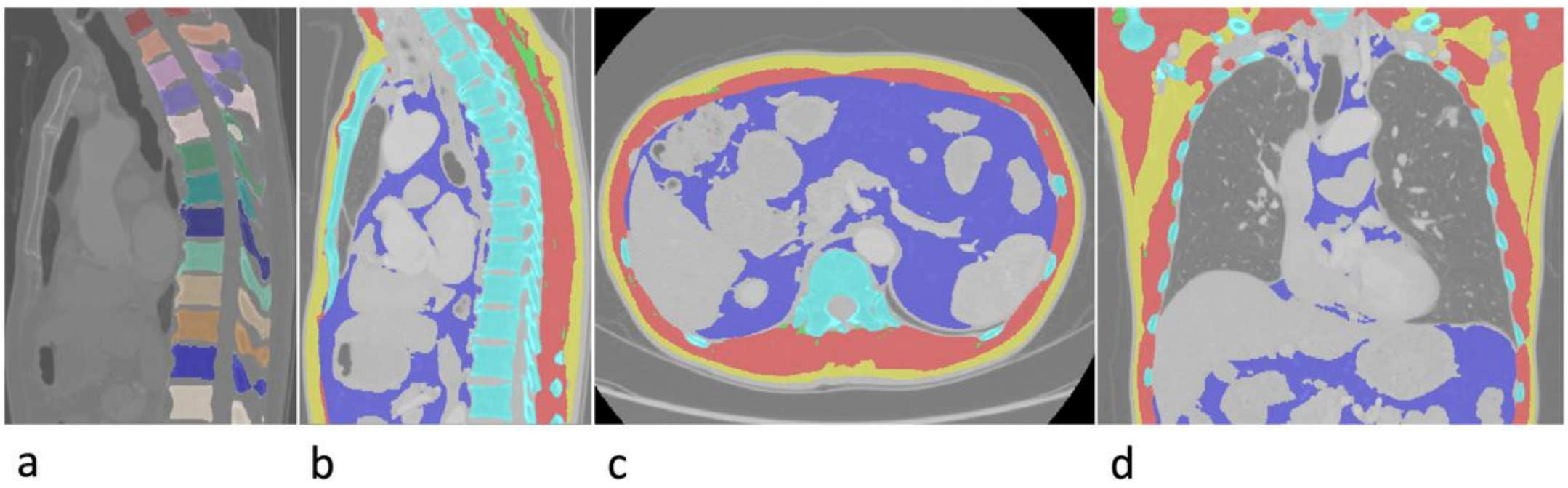

Fig. S4 Representative visualizations of automated spine localization and body composition segmentation. (a) Automated localization of vertebral bodies. (b-d) Exemplary results of automated segmentation for the five body composition components.

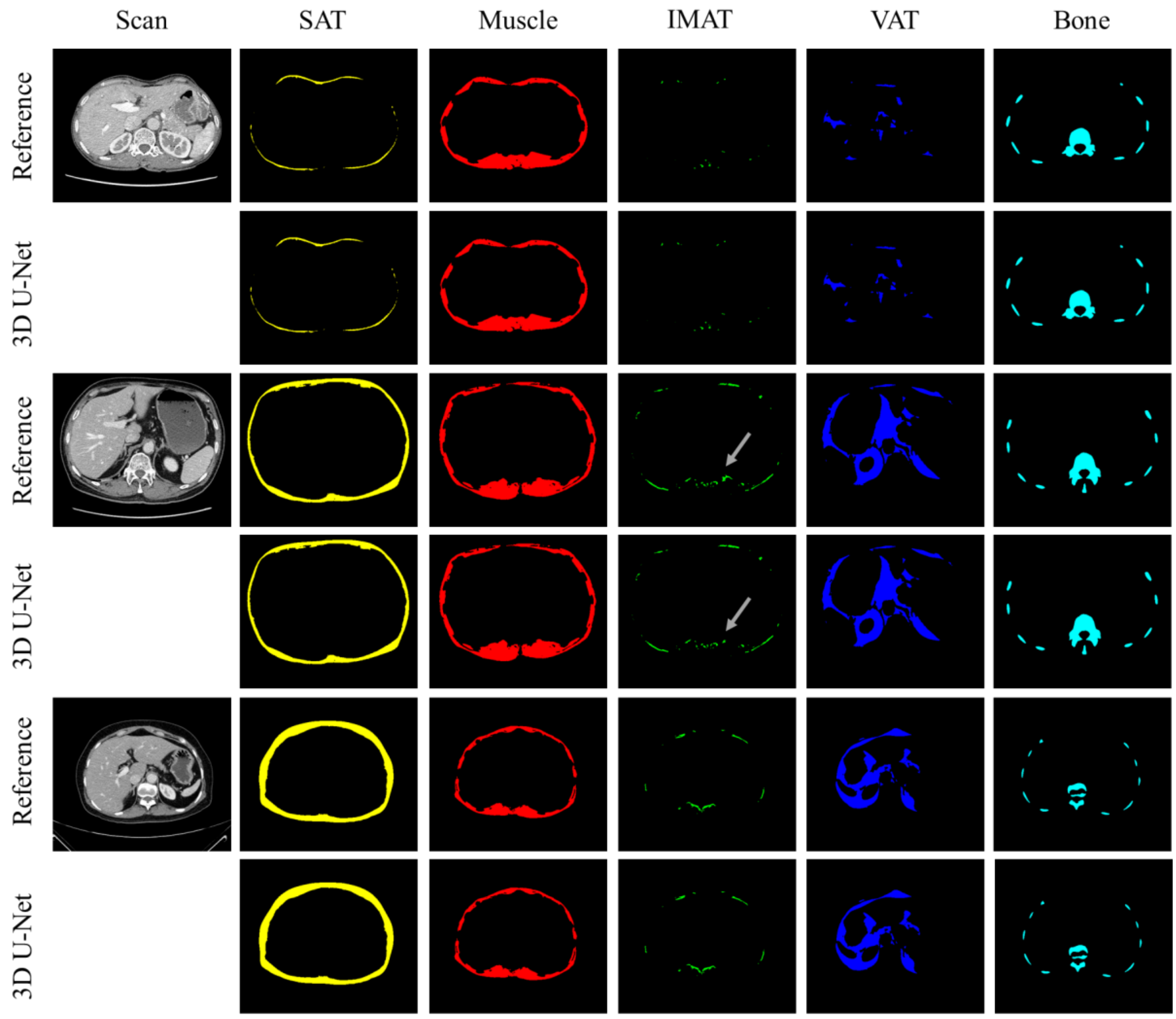

Fig. S5 Representative cases of automatic body composition segmentation. The original images (scans) are followed by images of SAT, muscle, IMAT, VAT, and bone. For each scan, the first row shows the segmentation using the manual method (Reference); the second row shows the prediction by 3D nnU-Net. Arrows indicate areas of annotation differences. Abbreviations: SAT, subcutaneous adipose tissue; IMAT, intermuscular adipose tissue, VAT, visceral adipose tissue.

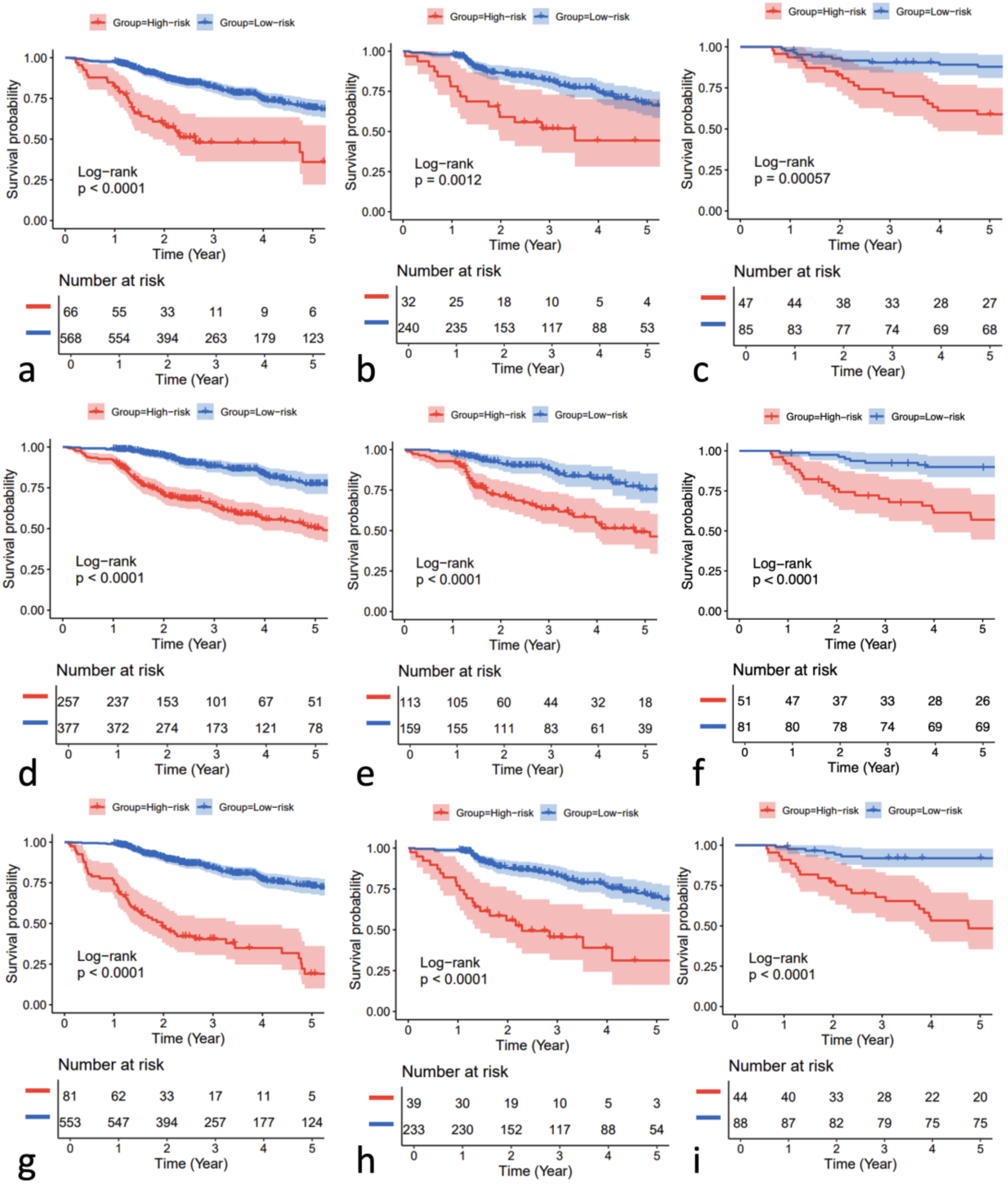

Fig. S6 Kaplan–Meier curves of overall survival based on body composition and tumor lesion Cox regression models. (a-c) Tumor radiomics (T-Rad) model. (d-f) Body composition radiomics (BC-Rad) model. (g-i) Integrated tumor and body composition (TB-Rad) model. (a, d, g) Training cohort. (b, e, h) Internal validation cohort. (c, f, i) External validation cohort.

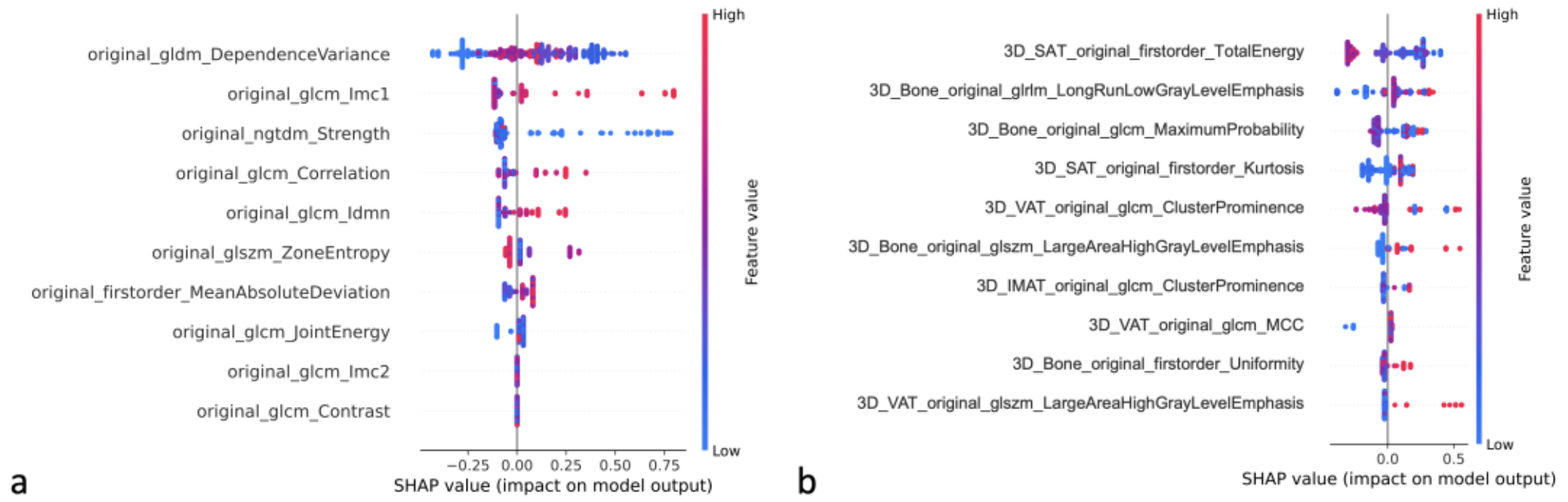

Fig. S7 SHAP beeswarm plots illustrating feature importance in XGBoost models. (a) Model based on tumor radiomics features. (b) Model based on body composition radiomics features.

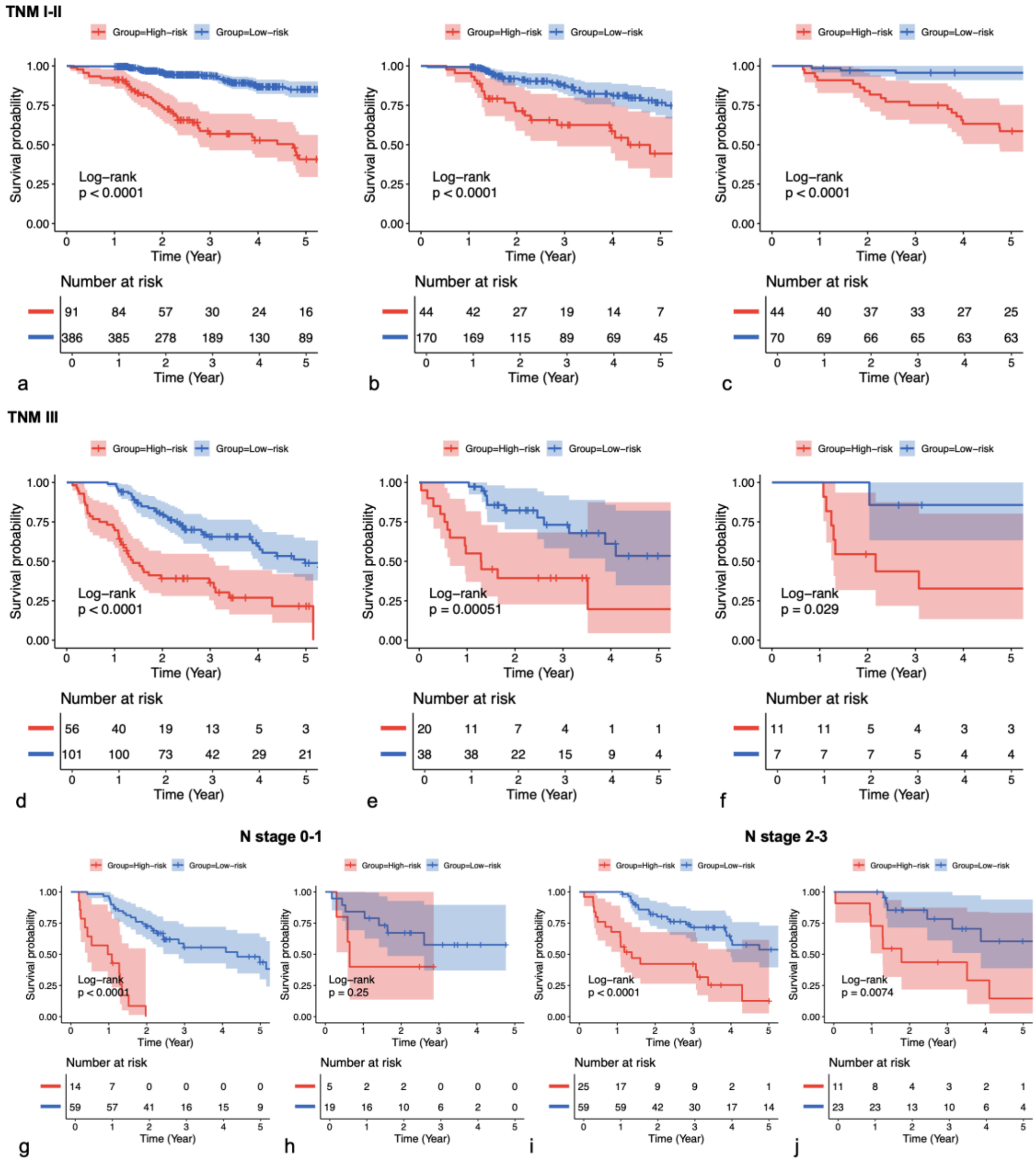

Fig. S8 Kaplan–Meier overall survival curves stratified by the CTB model in TNM subgroups and N-stage subgroups of TNM stage III. (a-c) TNM I-II stage. (d-f) TNM III stage. (g-h) N stage 0-1. (i-j) N stage 2-3. (a, d, g, i) Training cohort. (b, e, h, j) Internal validation cohort. (c, f) External validation cohort. CTB model, model integrating clinical variables, tumor radiomics score, and body composition radiomics score.

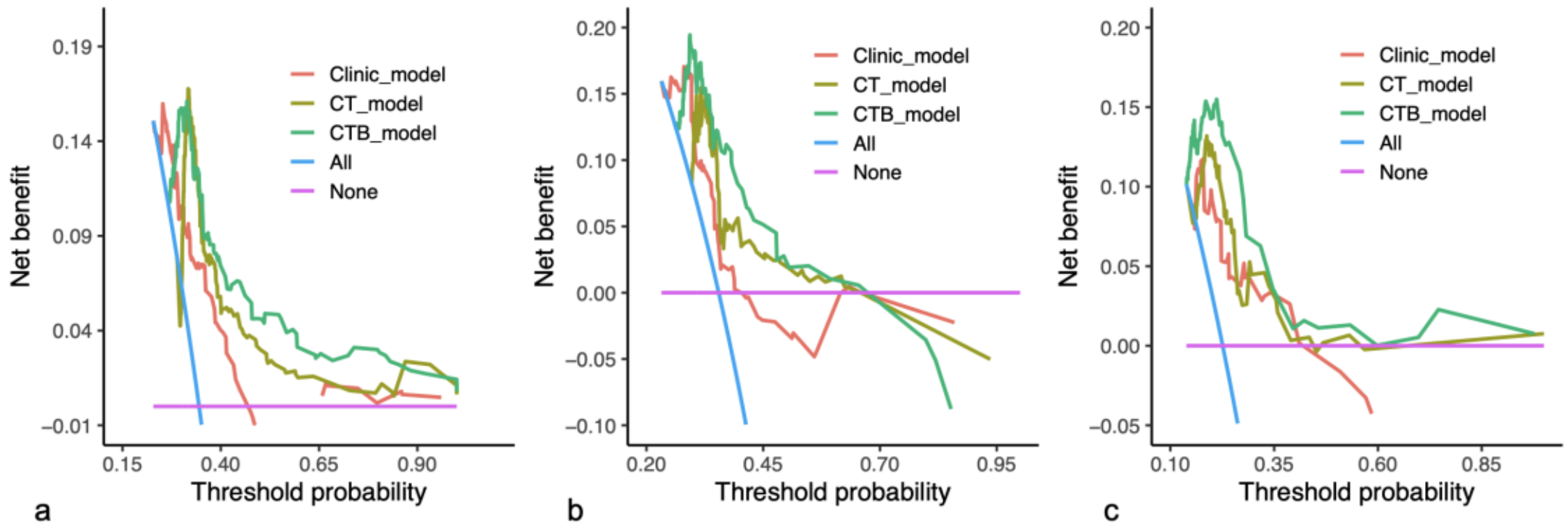

Fig. S9 Decision curve analysis evaluating the clinical utility of the predictive models. (a) Training cohort. (b) Internal validation cohort, and (c) External validation cohort.

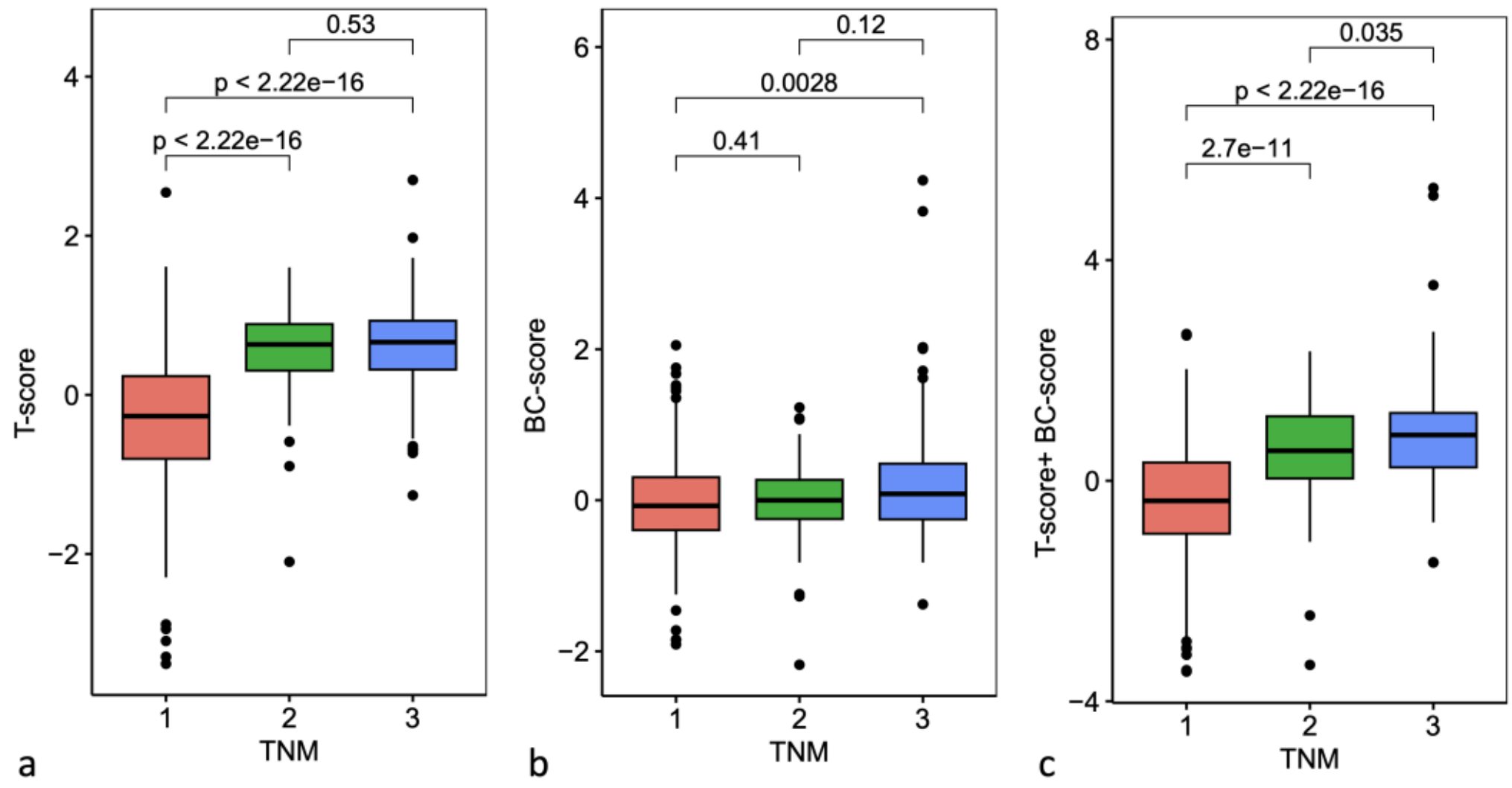

Fig. S10 Box plots of Tumor-score and Body composition-score in different TNM stages. (a) Tumor-score (T-score). (b) Body composition-score (BC-score). (c) Combined T-score and BC-score. Abbreviations: T-score, tumor-score; BC-score, body composition-score; TNM, tumor node metastasis.

Supplementary Tables

Table S1 CT scanning parameters.

Parameters	Center A	Center B	Center C
Manufacturers	Siemens (Somatom Definition AS or Biograph 40); GE (LightSpeed 8 or VCT 64); Philips (Brilliance iCT 256)	Toshiba (Aquilion One 320), Siemens (Somatom Definition Flash 128), Philips (Brilliance, 64)	Siemens (SOMATOM Emotion 16; SOMATOM Definition AS 128; SOMATOM Definition Flash)
Tube voltage	120 kV	100-120 kV	120-140 kV
Tube current	72-693 mAs	77-470 mAs	230-694 mAs
Slice thickness	1.0-2.5 mm	1.0-3.0 mm	1.0-3.0 mm
Data matrix	512×512	512×512	512×512
Pixel spacing	0.607-0.751 mm	0.625 mm	0.625 mm
Contrast agent concentration	300 mgI/ml	300 mgI/ml	300 mgI/ml
Contrast agent dosage	1.5 mg/kg	1.5 mg/kg	1.5 mg/kg
Contrast agent infused rate	3.5(ml/s)	3.0 or 3.5 ml/s	3.0 ml/s
Venous phase	60 s after injection	60 s after injection	65 s after injection

Table S2 Performance of the body composition automatic segmentation model in the internal validation cohort.

Body composition	Total	SM	IMAT	VAT	SAT	Bone
Dice	98.39%	99.66%	90.47%	99.67%	99.24%	98.88%
F1 score	98.95%	99.71%	86.34%	99.95%	99.29%	98.91%
JS	96.87%	99.33%	86.34%	99.34%	98.53%	97.81%
Sensitivity	98.95%	99.98%	78.65%	99.98%	99.97%	99.94%
Specificity	99.96%	86.50%	99.99%	99.99%	99.99%	99.94%
Positive predictive value	98.95%	99.43%	99.46%	99.61%	99.61%	97.88%
Negative predictive value	98.95%	99.47%	99.84%	99.95%	99.95%	98.91%

Abbreviations: SM, skeletal muscle; IMAT, intermuscular adipose tissue; VAT, visceral adipose tissue; SAT, subcutaneous adipose tissue; JS, jaccard score.

Table S3 Performance of the body composition segmentation model in the external validation cohort.

Body composition	Total	SM	IMAT	VAT	SAT	Bone
Dice	97.81%	99.92%	89.13%	99.95%	99.99%	99.98%
F1 score	98.42%	99.82%	86.13%	99.72%	99.99%	99.91%
JS	96.41%	99.72%	84.31%	99.24%	98.99%	99.21%
Sensitivity	98.45%	99.99%	72.99%	99.94%	99.999%	99.42%
Specificity	99.45%	90.70%	99.99%	99.99%	99.99%	99.82%
Positive predictive value	98.41%	99.72%	99.21%	99.62%	99.99%	99.21%
Negative predictive value	98.75%	99.86%	99.75%	99.92%	99.99%	99.91%

Abbreviations: SM, skeletal muscle; IMAT, intermuscular adipose tissue; VAT, visceral adipose tissue; SAT, subcutaneous adipose tissue; JS, jaccard score.

Table S4 Correlation and consistency analysis of the segmentation of body composition by manual and automated model.

Body composition	*r*	*P* value	ICC	95% CI
Skeletal muscle	0.999	<0.001	0.999	0.998, 1.000
Intermuscular adipose tissue	0.999	<0.001	0.998	0.997, 0.998
Visceral adipose tissue	0.999	<0.001	0.999	0.998, 1.000
Subcutaneous adipose tissue	0.999	<0.001	0.999	0.998, 1.000
Bone	0.999	<0.001	0.999	0.998, 1.000

Abbreviations: ICC, interclass correlation coefficient; CI, confidence interval.

Table S5 Survival prediction performance of radiomics-based models.

Models	Training cohort		Internal validation cohort		External validation cohort	
	C-index (95% CI)	*p* value	C-index (95% CI)	*P* value	C-index (95% CI)	*P* value
Cox regression						
T-Rad	0.719 (0.640, 0.798)	-	0.683 (0.572, 0.794)	-	0.705 (0.551, 0.839)	-
BC-Rad	0.653 (0.561, 0.745)	0.032	0.640 (0.499, 0.781)	0.181	0.648 (0.476, 0.820)	0.138
TB-Rad	0.752 (0.714, 0.790)	<0.001	0.722 (0.665, 0.779)	0.012	0.738 (0.654, 0.822)	0.019
XGBoost						
T-Rad	0.728 (0.624, 0.792)	-	0.695 (0.558, 0.785)	-	0.692 (0.564, 0.813)	-
BC-Rad	0.682 (0.568, 0.727)	0.026*	0.671 (0.525, 0.762)	0.482	0.674 (0.498, 0.807)	0.423
TB model	0.765 (0.729, 0.826)	<0.001*	0.749 (0.687, 0.811)	<0.001*	0.742 (0.663, 0.821)	<0.001*

NOTE: *Compared with T-Rad, $p < 0.05$. Abbreviations: CI, confidence interval; T-Rad, tumor radiomics; BC-Rad, body composition radiomics; TB-Rad, model integrating tumor and body composition radiomics.
